# Primary Cilia Formation Mediated by Hsa_Circ_0005185/OTUB1/RAB8A Complex Inhibits Prostate Cancer Progression by Suppressing Hedgehog Signaling Pathway

**DOI:** 10.1002/advs.202411675

**Published:** 2025-01-09

**Authors:** Aoyu Fan, Yunyan Zhang, Yunpeng Li, Wei Meng, Fan Wu, Wei Pan, Zhongliang Ma, Wei Chen

**Affiliations:** ^1^ Department of Urology Zhongshan Hospital Fudan University Shanghai 200030 China; ^2^ Lab for Noncoding RNA and Cancer School of Life Sciences Shanghai University Shanghai 200444 China

**Keywords:** circular RNAs, deubiquitination, hedgehog signaling, primary cilia, prostate cancer, Otubain 1 (OTUB1)

## Abstract

Prostate cancer (PCa) is one of the most common malignancies for male individuals globally. Androgen deprivation therapy (ADT) initially demonstrated significant efficacy in treating PCa; however, most cases of PCa eventually progress to castration‐resistant prostate cancer (CRPC), which becomes increasingly challenging to manage. Notably, the loss or disruption of primary cilia in PCa cells may play a critical role in the progression of the disease, and there are no reports on the role of circular RNAs in ciliogenesis. Thus, this warrants further investigation.In this study, key circular RNAs linked to prostate cancer progression, and enzalutamide resistance is identified. Specifically, it is found that hsa_circ_0005185 interacts with OTUB1 and RAB8A, serving as a molecular scaffold. Hsa_circ_0005185 mediates the binding of the deubiquitinase OTUB1 to RAB8A, resulting in the deubiquitination of RAB8A. Consequently, the stable expression of RAB8A promotes the regeneration of primary cilia and enhances the production of GLI3R, an inhibitory factor in the Hedgehog signaling pathway, thereby suppressing AR activity and slowing the progression of CRPC.

## Graphical abstract

Circular RNA hsa_circ_0005185 functions as a molecular scaffold, facilitating the interaction between OTUB1 and RAB8A, leading to the deubiquitination of RAB8A. This process promotes primary cilia formation and enhances GLI3R production in the Hedgehog signaling pathway, ultimately inhibiting androgen receptor activity and suppressing the progression of prostate cancer.

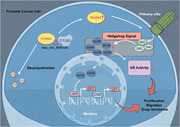

## 1 Introduction

Prostate cancer remains a major global concern in men's health.^[^ ^1^ ^]^ Androgen deprivation therapy (ADT) plays a central role in its treatment; however, as the disease advances, hormone‐sensitive prostate cancer frequently transitions into castration‐resistant prostate cancer (CRPC).^[^ ^2^ ^]^ For many years, research into the formation mechanism of CRPC has primarily focused on the androgen signaling pathway.^[^ ^3^, ^4^ ^]^ Building on these findings, new drugs such as abiraterone and enzalutamide have been developed and widely adopted, demonstrating improved efficacy and extending patient survival.^[^ ^5^ ^]^ Despite their success, disease‐free survival with these treatments is limited, emphasizing the need for further exploration of drug resistance mechanisms and the development of novel therapeutic strategies in prostate cancer research.

Circular RNAs (circRNAs) are non‐coding RNAs produced by back‐splicing of a precursor mRNA transcript to form a covalently closed continuous loop without a 5′ cap or 3′ poly‐A tail.^[^ ^6^ ^]^ Due to their unique biochemical structure, circRNAs are far more stable in cells and less susceptible to digestion by RNase R. The biological functions of circRNAs in prostate cancer have been widely reported.^[^ ^7^, ^8^ ^]^ Increasing evidence suggests that circRNAs are involved in prostate cancer proliferation, invasion, and drug resistance.^[^ ^9^ ^]^ Currently, the relationship between circRNAs and drug resistance in prostate cancer is poorly understood. In this study, we performed circRNA sequence screening on CRPC and hormone‐sensitive prostate cancer (HSPC), to identify circular RNAs associated with CRPC development and further investigate their regulatory mechanisms.

In recent years, studies have found that the absence of primary cilia is closely related to the progression and invasiveness of prostate cancer.^[^ ^10^, ^11^ ^]^ In hormone‐related tumors, primary cilia, as important organelles, can mediate the regulation of hormone signaling pathways on the fate of tumor cells.^[^ ^12^, ^13^ ^]^ If specific circular RNAs can be applied to regulate the regeneration of primary cilia, they may pave the way for the study of CRPC. Primary cilia are microtubule‐based organelles that protrude from the surface of most mammalian cells and are distinct from motile cilia and primarily function as signaling agents that transduce extracellular signals to the cell body.^[^ ^14^ ^]^ Initially studied in the context of genetic diseases known as ciliopathies, recent research has expanded to reveal that defects in primary cilia are implicated in various cancers, including pancreatic ductal adenocarcinoma, renal cell carcinoma, cholangiocarcinoma, glioblastoma, basal cell carcinoma, and prostate cancer.^[^ ^12^, ^15^ ^]^ Despite the identification of numerous ciliary structural components and regulators, the mechanisms by which primary cilia dysfunction contributes to cancer remain unclear. In this study, we explored the potential role of circ_0005185 (hsa_circ_0005185) in ciliogenesis in prostate cancer, intending to establish its contribution to ciliary loss in this disease. Primary cilia are associated with several signaling pathways, including the Wnt, Hedgehog (Hh), and PDGFRα pathways, with the Hh signaling pathway being particularly relevant.^[^ ^15^, ^16^, ^17^ ^]^ Primary cilia act as bifacial regulators of Hh signaling and are essential for Hh pathway activation. Phosphorylated Smoothened (Smo) activates the Gli family of transcription factors (GLI1, GLI2, GLI3) upon translocation to the cilium, whereas primary cilia facilitate the hydrolysis of GLI2 and GLI3 into their repressor forms (GLI2R, GLI3R).^[^ ^17^ ^]^ The Hh pathway plays a critical role in prostate cancer progression and the transition to CRPC.^[^ ^13^ ^]^ Research indicates that stromal cells can enhance acquired intratumoral steroidogenesis (AIS) through a paracrine Hh signaling environment.^[^ ^18^ ^]^ Therefore, targeting the Hh signaling pathway may represent a strategic approach to counteract stromal cell‐derived AIS and delay the onset of CRPC following androgen deprivation therapy. However, acquired resistance to Hh pathway inhibition has been observed in other cancers after prolonged treatment. Thus, exploring novel strategies for inhibiting Hh signaling in prostate cancer and other diseases could enhance its therapeutic potential.

Ciliogenesis and the function of primary cilia can be regulated by small RAB proteins.^[^ ^19^ ^]^ RAB8A, in particular, is crucial for cilia formation as it interacts with various proteins associated with the ciliary basal body and the ciliary axoneme/membrane. RAB8A is localized to the basal body, and its GTP‐bound form enters the primary cilia to facilitate the extension of the ciliary membrane.^[^ ^19^, ^20^ ^]^ Although primary cilia are key sites for activating the Hh signaling pathway, the specific role of RAB8A in regulating this pathway remains unclear and has not yet been documented due to the complex nature of the ciliary regulation of Hh signaling. OTUB1, a member of the ovarian tumor subfamily of de‐ubiquitinating enzymes (DUBs), plays a significant role in controlling protein stability and activity by negatively regulating ubiquitination. OTUB1 is known to be critical in various cellular processes, including DNA damage response, apoptosis, proliferation, and cancer development.^[^ ^21^, ^22^, ^23^ ^]^ However, the potential interaction between OTUB1 and RAB8A and the mechanism by which OTUB1 promotes RAB8A deubiquitination have not been reported.

## 2 Results

### 2.1 The Expression and Clinical Features of Circ_0005185 in PCa Tissues

**Figure 1:**
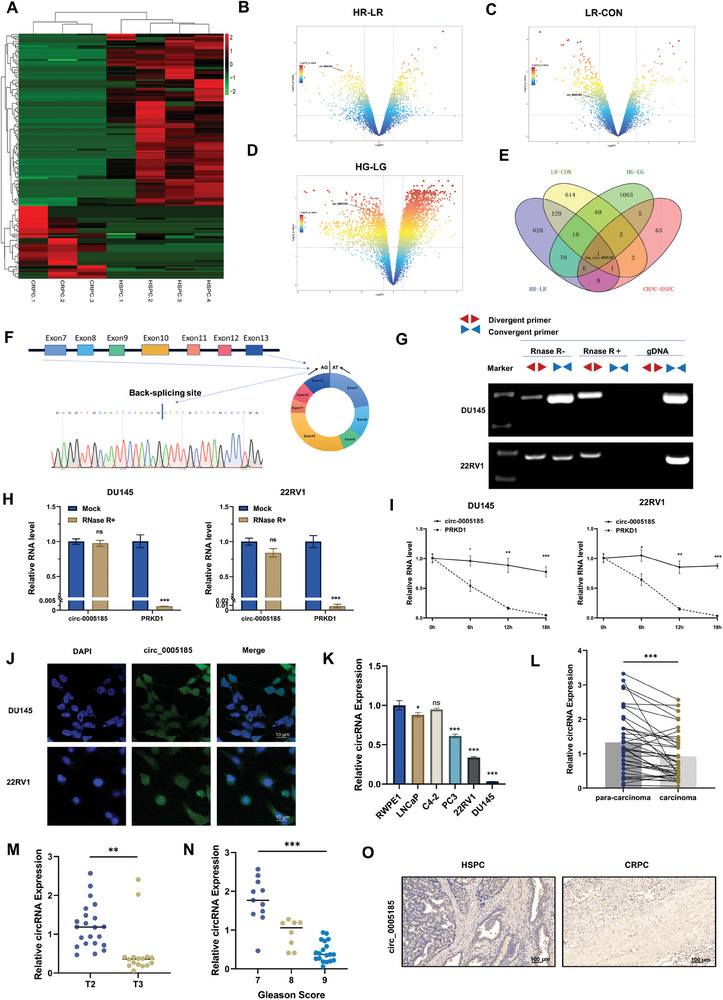
circ_0005185 is identified as a circular RNA that is downregulated in castration‐resistant prostate cancer (CRPC) tissues, high Gleason score prostate cancer (PCa) tissues, and enzalutamide‐resistant PCa cell lines. A) A hierarchical clustering heatmap displays the circular RNAs with the most significant expression differences between HSPC and CRPC tissues. Red in the heatmap represents upregulation. Green represents downregulation. B,C) Volcano plots show the differential gene analysis between the high‐resistance (HR) and low‐resistance (LR) groups to enzalutamide, as well as the low‐resistance (LR) group and the control (CON) group. D) Volcano plot displays the differential gene analysis between the high Gleason (HG) score group and the low Gleason (LG) score group. E) The Venn diagram of differentially expressed circRNAs across the four groups (HR‐LR, LR‐CON, HG‐LG, CRPC‐HSPC) reveals that circ_0005185 is downregulated in CRPC, high Gleason score prostate cancer, and enzalutamide‐resistant cell lines. F) Genomic location and splicing pattern of circ_0005185. The splice junction sites were confirmed by Sanger sequencing. G) Agarose gel electrophoresis following RT‐PCR demonstrates the stability and back‐splicing of circ_0005185 in DU145 and 22RV1 cell lines. Divergent primers amplify the junction site of circ_0005185 in cDNA but not in genomic DNA (gDNA), confirming the circular nature of circ_0005185. H) The qRT‐PCR results show the abundance of circ_0005185 and PRKD1 mRNA in DU145 and 22RV1 cells after RNase R treatment. I) The qRT‐PCR results of circ_0005185 and PRKD1 mRNA abundance at various time points after treatment with actinomycin D in DU145 and 22RV1 cells reveal insights into their stability profiles. J) The localization of circ_0005185 in DU145 and 22RV1 cells was investigated using a FAM‐labeled (green) probe specific to circ_0005185, while the cell nuclei were stained with DAPI (blue). The scale bar represents 20 µm. K) The expression levels of circ_0005185 were determined using qRT‐PCR in five prostate cancer cell lines (LNCaP, 22RV1, DU145, PC3, C4‐2) and normal prostate epithelial cells (RWPE1). L) The expression of circ_0005185 was validated in 38 pairs of prostate cancer tissues and adjacent non‐tumor tissues. M) Expression of circ_0005185 in patients with stage T2 and T3. N) Expression of circ_0005185 in patients with Gleason score of 7, 8, and 9. O) The expression abundance of circ_0005185 in HSPC and CRPC was determined by in situ hybridization (scale bar = 100 µm). Data are presented as the mean ± SD (^*^ *p* < 0.05; ^**^ *p* < 0.01; ^***^ *p* < 0.001, ns, not significant).

To identify circRNAs associated with prostate cancer progression and drug resistance, we performed circRNA sequencing on four HSPC and three CRPC tissues (**Figure** **1**A). Additionally, circRNA microarray data were retrieved from the GEO database, focusing on prostate cancer tissues with varying Gleason scores, as well as LNCaP cell lines exhibiting different levels of resistance to enzalutamide. Subsequently, we conducted a tiered analysis of differentially expressed genes, focusing on the gradients of enzalutamide resistance. Specifically, we analyzed the differentially expressed circRNAs between the high‐ and low‐resistance groups, and between the low‐resistance and control groups (Figure 1B,C). Finally, we analyzed the differentially expressed circRNAs between the high and low Gleason score groups (Figure 1D). Our findings revealed that circ_0005185 was significantly downregulated in both CRPC and high Gleason score tissues, as well as in cell lines demonstrating high enzalutamide resistance (Figure 1E). circ_0005185 is a sense‐overlapping circular transcript derived from exons 7–13 of the PRKD1 gene. Sanger sequencing was used to confirm the head‐to‐tail splicing of circ_0005185 (Figure 1F). To characterize the loop structure of circ_0005185, divergent primers were designed to amplify its loop‐forming sites, alongside convergent primers for PRKD1. Our templates consisted of complementary DNA (cDNA) and genomic DNA (gDNA) extracted from DU145 and 22RV1 cells and one set of cDNAs treated with RNase R. As expected, the divergent primers successfully amplified circ_0005185 using cDNA (but not gDNA) as a template, regardless of RNase R treatment. Conversely, PRKD1 mRNA could not be amplified from cDNA treated with RNase R. Furthermore, the qRT‐PCR results indicated that RNase R did not significantly affect circ_0005185 expression (Figure 1G,H). The actinomycin D assay demonstrated that circ_0005185 levels remained relatively stable after treatment, whereas PRKD1 mRNA levels decreased over time (Figure 1I). Collectively, these findings support the circular nature of circ_0005185 and confirm its presence in prostate cancer cells. Fluorescence in situ hybridization (FISH) assays revealed that circ_0005185 was predominantly localized in the cytoplasm of DU145 and 22RV1 cells (Figure 1J). Subsequently, we examined the expression of circ_0005185 in prostate cancer cell lines (LNCaP, 22RV1, DU145, PC3, C4‐2) and normal prostate epithelial cells (RWPE1). The results showed that circ_0005185 was lowly expressed in prostate cancer cells, with significant downregulation observed in PC3, DU145, and 22RV1 cells (Figure 1K). Finally, we validated the expression of circ_0005185 in 38 pairs of prostate cancer tissue samples and adjacent normal tissues, revealing that circ_0005185 is expressed at lower levels in prostate cancer specimens (Figure 1L). The clinicopathological features of 38 patients are shown in Table S1(Supporting Information). According to the relative expression of circ_0005185 in PCa tissues, the patients were divided into high expression group and low expression group. As shown in Table S1 (Supporting Information), low circ_0005185 expression was significantly associated with a higher T stage (*p* < 0.001), higher N stage (*p* = 0.042), and higher Gleason Score (*p* < 0.001). In addition, the expression of circ_0005185 in stage T3 patients was significantly lower than that in stage T2 patients (Figure 1M ). circ_0005185 was detected in different Gleason Score groups, and the results showed that the expression of circ_0005185 was lower in the group with a higher Gleason score (Figure 1N). Finally, we used in situ hybridization to detect the abundance of circ_0005185 in HSPC and CRPC tissues, which showed a low abundance in CRPC tissues (Figure 1O).

### 2.2 Circ_0005185 Inhibits Prostate Cancer Proliferation, Migration, and Enzalutamide Resistance

**Figure 2:**
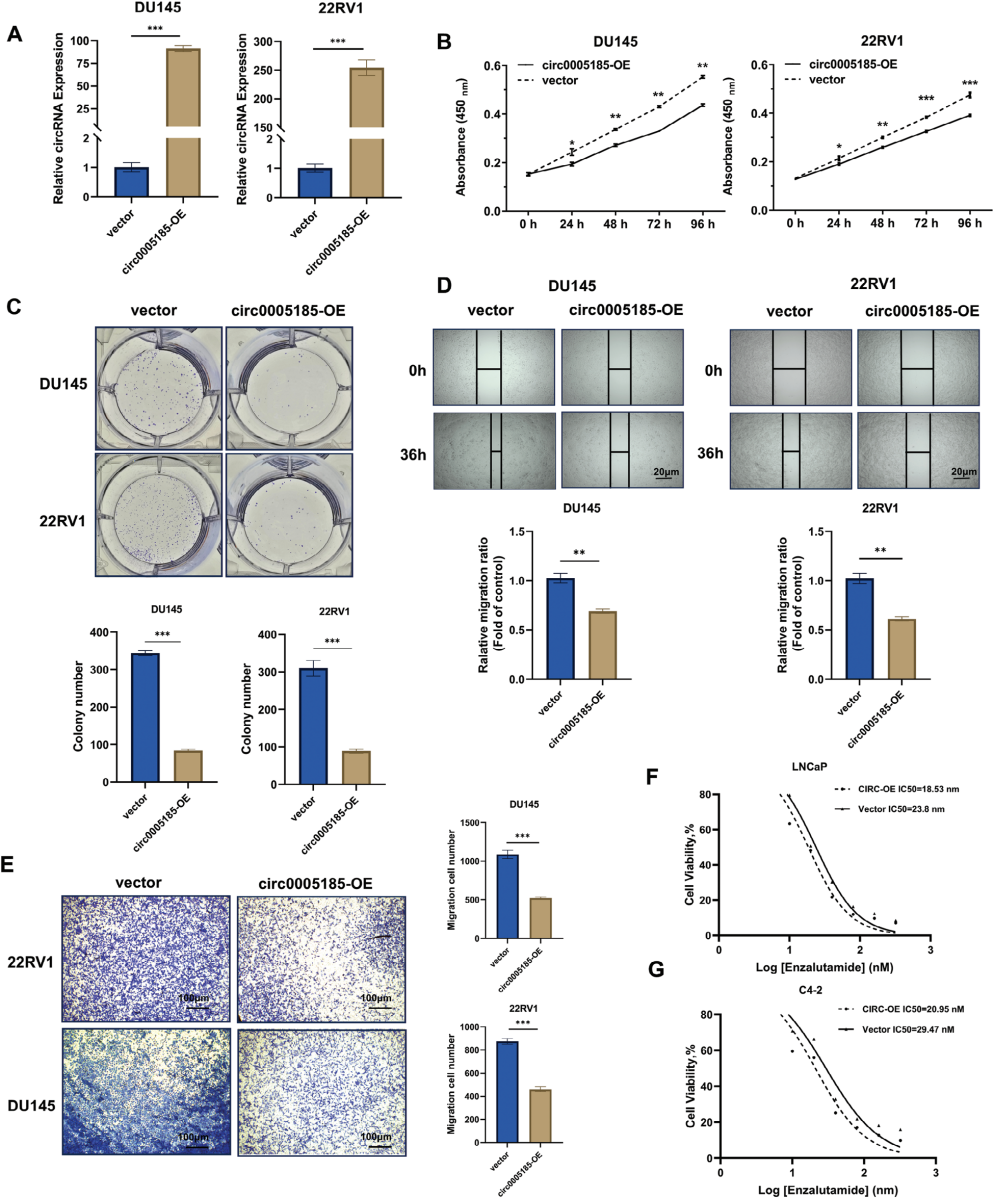
circ_0005185 inhibits prostate cancer proliferation, migration, and enzalutamide resistance. A) qRT‐PCR validation of circ_0005185 overexpression efficiency. B) CCK8 assay showing that overexpression of circ_0005185 inhibits proliferation in DU145 and 22RV1 cells. C) Colony formation assay and statistical analysis confirming the inhibitory effect of circ_0005185 overexpression on proliferation in DU145 and 22RV1 cells. D) Scratch wound healing assay demonstrated that overexpression of circ_0005185 inhibits migration in DU145 and 22RV1 cells (scale bar = 20 µm). The relative migration ratio is displayed in the lower panel. E) Transwell migration assay demonstrating that overexpression of circ_0005185 inhibits migration in DU145 and 22RV1 cells (scale bar = 100 µm). The numbers of migration cells are displayed in the right panel. F‐G) Measurement of enzalutamide IC50 values in LNCaP and C4‐2 cells after overexpression of circ_0005185 compared to control cells. Data are presented as the mean ± SD (^*^ *p* < 0.05; ^**^ *p* < 0.01; ^***^ *p* < 0.001, ns, not significant).

To investigate the biological role of circ_0005185 in prostate cancer, lentiviral vectors were used to overexpress circ_0 005185 in DU145 and 22RV1 cells (**Figure** **2**A). The results of the CCK‐8 assay indicated that the overexpression of circ_0 005185 significantly inhibited the proliferation of these prostate cancer cell lines (Figure 2B). Similarly, colony formation assays demonstrated that circ_0 005185 reduced the clonogenic capacity of prostate cancer cells (Figure 2C). Furthermore, wound healing and transwell assays were conducted to evaluate the migratory potential of the cells. The results illustrated that the overexpression of circ_0 005185 notably reduced the migration ability of prostate cancer cells (Figure 2D,E). Given that enzalutamide‐resistant cell lines exhibited lower levels of circ_0 005185 expression, cytotoxicity assays were also performed to assess the impact of circ_0 005185 on the sensitivity of enzalutamide‐sensitive LNCaP and C4‐2 prostate cancer cells to enzalutamide. Our findings revealed that increasing circ_0 005185 levels effectively mitigated the resistance of prostate cancer cells to Enzalutamide (Figure 2F,G).

### 2.3 Circ_0005185 Binds to the 196–247 aa Region of OTUB1 and the 32–83 aa Region of RAB8A

**Figure 3:**
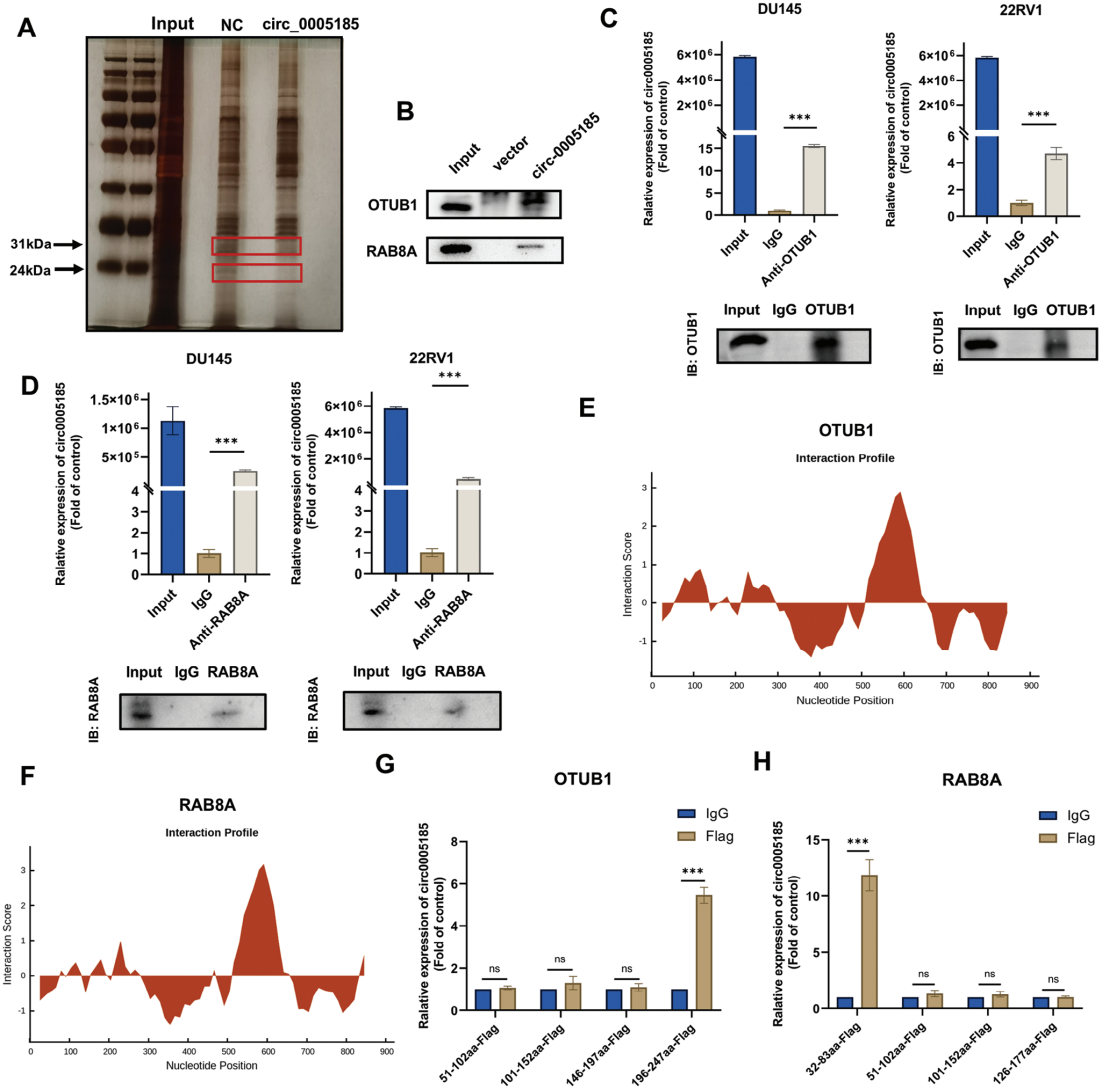
circ_0005185 binds to the 196–247 aa region of OTUB1 and the 32–83 aa region of RAB8A. A) Silver staining image of an SDS‐PAGE gel displaying the isolation of circ_0005185/protein complexes from the RNA pull‐down experiment in DU145 cells; the red box highlights the specific protein bands enriched in the pull‐down complex by the circ_0005185 probe compared to the NC probe. B) RNA pull‐down assays coupled with western blot analysis confirmed the binding of circ_0005185 to OTUB1 and RAB8A proteins. C,D) RIP experiments utilizing OTUB1 and RAB8A primary antibodies or IgG were performed to assess the enrichment of circ_0005185 with proteins in DU145 and 22RV1 cells. Western blot was used to detect OTUB1 and RAB8A proteins immunoprecipitated by their respective antibodies or IgG. E–F) Interaction profiles of various regions of OTUB1 and RAB8A proteins with circ_0005185 were analyzed using the catRAPID database (http://www.tartaglialab.com/) to gain insights into their binding specificity. G) RIP assays were employed to quantitatively determine the enrichment of circ_0005185 within the 51–102, 101–152, 146–197, and 196–247 amino acid (aa) regions of the OTUB1 protein. H) Similarly, RIP assays were employed to quantitatively assess the enrichment of circ_0005185 within the 32–83, 51–102, 101–152, and 126–177 aa regions of the RAB8A protein. Data are presented as the mean ± SD (^*^ *p* < 0.05; ^**^ *p* < 0.01; ^***^ *p* < 0.001, ns, not significant).

To investigate the molecular mechanisms by which circ_0 005185 influences prostate cancer progression, we conducted RNA pull‐down assays, silver staining, and mass spectrometry to identify proteins that bind to circ_0005185. The mass spectrometry analysis revealed that circ_0005185 can bind to OTUB1 and RAB8A when compared to the control group. Additionally, SDS‐PAGE gel electrophoresis displayed significant bands at 24 and 31 kDa in the circ_0005185 probe group, contrasting with those in the control group (**Figure** **3**A). The western blotting results further confirmed that the proteins isolated from circ_0005185 included both OTUB1 and RAB8A (Figure 3B). To validate the binding of the OTUB1 and RAB8A proteins to circ_0005185, we performed RIP experiments. Nonspecific results were excluded using western blotting, which demonstrated successful binding of the OTUB1 and RAB8A antibodies to magnetic beads (Figure 3C,D), suggesting a binding relationship between circ_0005185, OTUB1, and RAB8A. Next, we used the CatRAPID database to predict specific regions of interaction between RAB8A and OTUB1 with circ_0005185. The predictions indicated that circ_0 005185 is likely to interact with the 51–102, 101–152, 146–197, and 196–247 AA regions of the OTUB1 protein, as well as the 32–83, 51–102, 101–152, and 126–177 amino acid (AA) regions of the RAB8A protein (Figure 3E,F). Based on the predicted protein sequences, pCDNA3.1‐Flag plasmids were constructed corresponding to each of these regions. Subsequently, these plasmids were transfected into DU145 cells, and RIP experiments were conducted using an anti‐Flag antibody. The abundance of circ_0 005185 was quantified using qRT–PCR. The results illustrated that compared to the negative control group, the 196–247 aa‐Flag regions of OTUB1 and 32–83 aa‐Flag of RAB8A were significantly enriched in circ_0 005185, whereas the other fragments did not exhibit significant enrichment (Figure 3G,H). Finally, western blotting was performed to verify the binding of Flag antibodies to the beads, ensuring that nonspecific results in qRT‐PCR detection were ruled out (Figure S1A,B, Supporting Information). These findings suggest that circ_0 005185 interacts specifically with the 196–247 aa region of OTUB1 and the 32–83 aa region of RAB8A, highlighting their potential roles in mediating the effects of circ_0 005185 on prostate cancer progression.

### 2.4 circ_0005185 Facilitates the Deubiquitination at K48 Site of RAB8A by Mediating the Interaction Between OTUB1 and RAB8A

**Figure 4:**
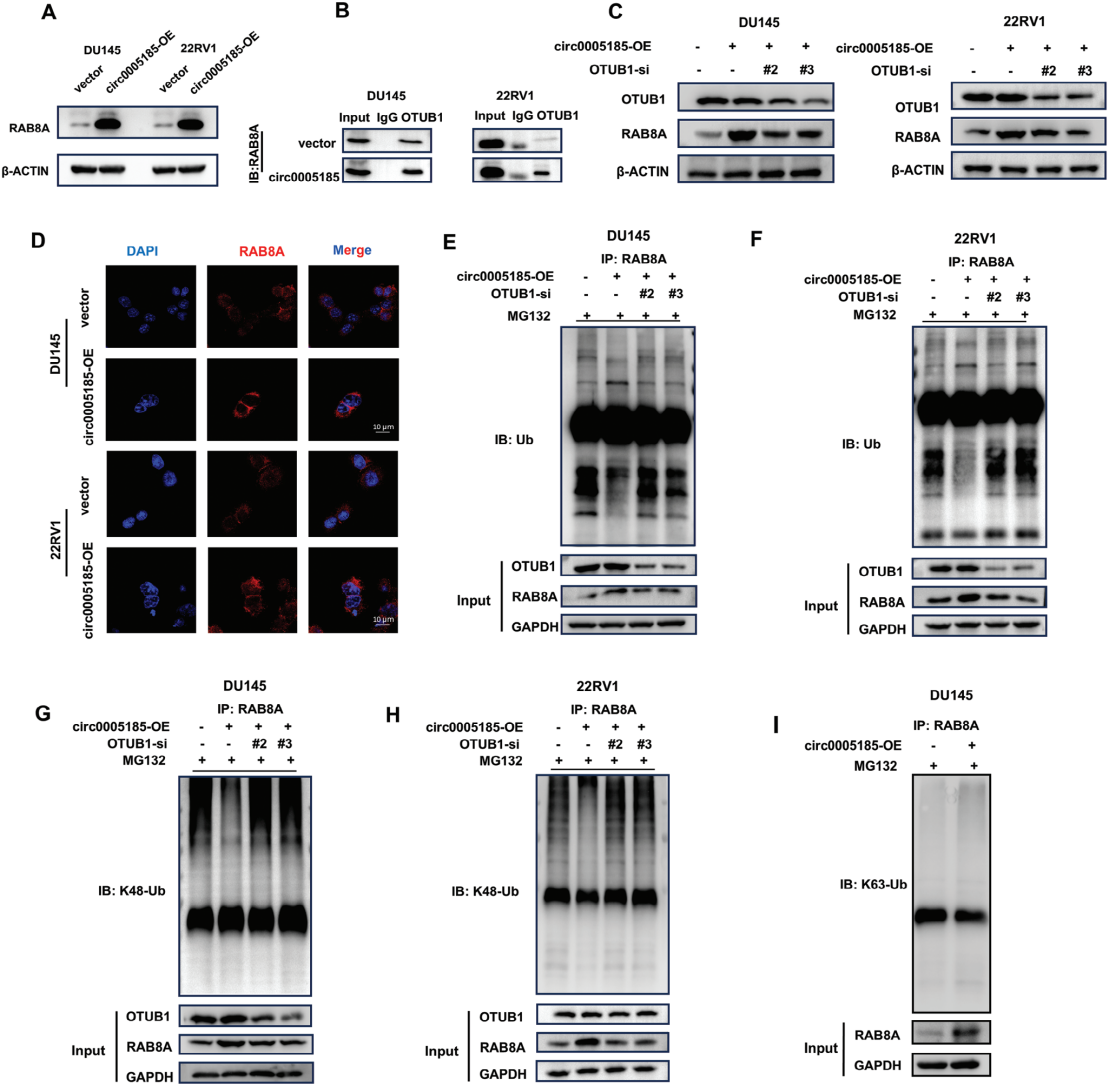
circ_0005185 facilitates the deubiquitination of RAB8A by mediating the interaction between OTUB1 and RAB8A. A) Western blot showed that the protein level of RAB8A increased after overexpression of circ_0005185. B) The Co‐IP experiment used OTUB1 antibody to verify the binding between RAB8A and OTUB1, which increased after overexpression of circ_0005185. C) Western blot showed that the protein level of RAB8A increased after overexpression of circ_0005185, while knockdown of OTUB1 rescued the level of RAB8A. D) The results of immunofluorescence showed the localization and expression of RAB8A in the control group and circ_0005185 overexpression group. E,F) The ubiquitination level of RAB8A was detected in DU145 and 22RV1 cells using ubiquitination antibodies. Overexpression of circ_0005185 led to decreased ubiquitination of RAB8A, while knockdown of OTUB1 resulted in increased ubiquitination of RAB8A. G,H) The regulation of ubiquitination at the K48 site of RAB8A by OTUB1 was detected in DU145 and 22RV1 cells using antibodies specific to the K48 ubiquitination site. I) Ubiquitination at the K63 site of RAB8A was detected in DU145 cells using antibodies specific to the K63 ubiquitination site.

We then investigated whether circ_0 005185 overexpression influenced the protein expression of RAB8A and OTUB1. The results demonstrated that circ_0 005185 overexpression significantly increased the protein expression of RAB8A (**Figure** **4**A). Previous studies have indicated that the RAB family is regulated by the ubiquitin‐proteasome system. Thus, it is hypothesized that OTUB1 promotes the deubiquitination of RAB8A, thereby regulating its protein levels. Next, a Co‐IP assay was conducted to examine the potential interaction between OTUB1 and RAB8A. The results revealed that RAB8A was successfully detected by western blotting using an OTUB1 antibody along with magnetic beads to isolate the bound proteins (Figure 4B). We also assessed the effect of circ_0 005185 on the binding of OTUB1 to RAB8A. Co‐IP analysis indicated that the overexpression of circ_0 005185 enhanced the binding of OTUB1 to RAB8A (Figure 4B). Subsequently, we used siRNAs to knock down OTUB1 in DU145 and 22RV1 cells (Figure S1C, Supporting Information). Notably, when OTUB1 was knocked down in the context of circ_0 005185 overexpression, there was a corresponding decrease in RAB8A protein levels (Figure 4C). The immunofluorescence results also showed that the fluorescence signal of RAB8A was enhanced after circ_0 005185 overexpression (Figure 4D). Next, ubiquitinating antibodies were used to explore how circ_0 005185 and OTUB1 affect RAB8A ubiquitination. Our findings indicated that overexpression of circ_0 005185 led to a reduction in RAB8A ubiquitination, whereas knockdown of OTUB1 increased RAB8A ubiquitination (Figures 4E,F and S1D,E, Supporting Information). We also investigated the ability of OTUB1 to eliminate K48‐linked and K63‐linked ubiquitin chains from RAB8A using antibodies specific to the K48 and K63 ubiquitination sites. Our results showed that OTUB1 modulated ubiquitination at the K48 site of RAB8A, whereas no ubiquitination was detected at the K63 site (Figures 4G–I and S1F, Supporting Information).

Collectively, these results suggest that circ_0 005185 mediates the interaction between OTUB1 and RAB8A and promotes the deubiquitination of RAB8A. This process inhibits the degradation of RAB8A and increases its protein levels, highlighting the regulatory role of circ_0 005185 in this pathway.

### 2.5 Overexpression of circ_0005185 Promotes Cilia Formation and Blocks the Hedgehog Pathway by Increasing GLI3R

**Figure 5:**
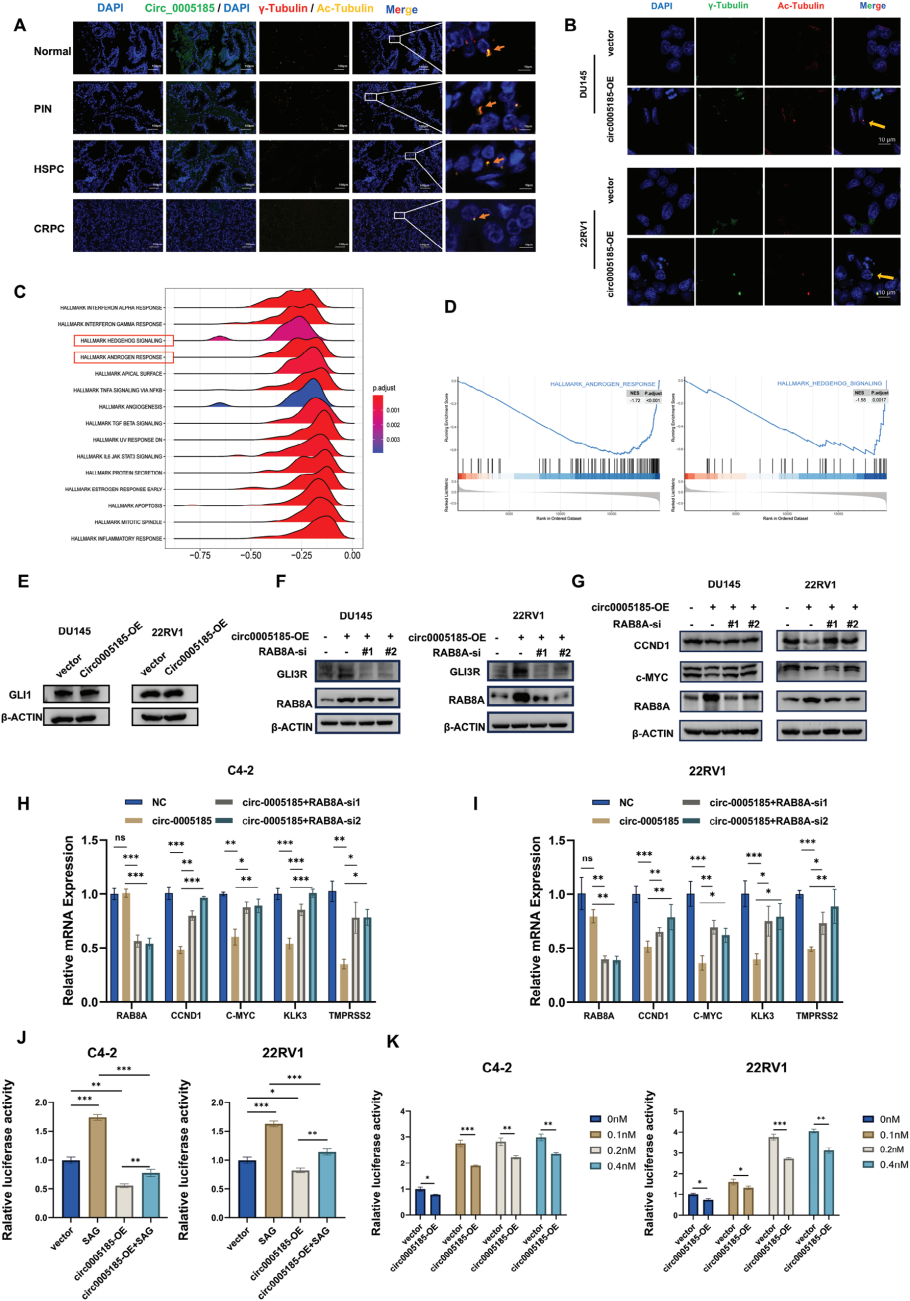
circ_0005185 promotes cilia formation and blocks the Hedgehog (Hh) pathway by increasing GLI3R. A) DAPI (blue), circ_0005185 (green), γ‐tubulin (red), and Ac‐tubulin (yellow) in normal prostate, Prostatic intraepithelial neoplasia (PIN), HSPC, and CRPC tumor samples were detected by multispectral IF staining and continuous FISH assay (Scale bar = 100 µm). Primary cilia morphology (yellow arrows) was observed at 10x magnification. B) Immunofluorescence detection of γ‐tubulin (green), Ac‐tubulin (red), and primary cilia (yellow arrows) in control and circ_0005185‐overexpressed DU145 and 22RV1 cells. Scale bar = 10 µm. C) Ridge plot from GSEA showing differences in AR response and Hh signaling pathway between patients with high and low RAB8A expression. D) GSEA of AR response and Hh signaling pathway. E) Protein expression of GLI1, a downstream activator of the Hh signaling pathway, was detected in the control and circ_0005185‐overexpressing cells. F) Protein levels of GLI3R, a downstream repressor of the Hh signaling pathway, were measured in control and circ_0005185‐overexpressing cells, as well as in circ_0005185‐overexpressing cells with RAB8A knockdown. G) The protein levels of RAB8A, CCND1, and c‐MYC were detected in control and circ_0005185‐overexpressing cells, as well as in circ_0005185‐overexpressing cells with RAB8A knockdown. H‐I) The expression of Hh signaling pathway target genes CCND1 and c‐MYC, as well as AR signaling pathway downstream target genes KLK3 and TMPRSS2, were examined in C4‐2 and 22RV1 cells after overexpression of circ_0005185. Additionally, the expression of these genes was assessed in circ_0005185‐overexpressing cells with RAB8A knockdown. J) The AR activity was measured in vector, SAG, circ0005185‐OE groups in C4‐2 and 22RV1 cells. The results were normalized with the vector group, respectively. K) The AR activity was measured in vector and circ0005185OE groups in C4‐2 and 22RV1 cells treated with EtOH, 0.1, 0.2, and 0.4 nm DHT for 24 h. Data are presented as the mean ± SD (^*^ *p* < 0.05; ^**^ *p* < 0.01; ^***^ *p* < 0.001, ns, not significant).

Small Rabs regulate the formation and function of primary cilia. RAB8A can be activated by RAB11A and plays an essential role in cilia formation and function by interacting with numerous proteins linked to ciliary basal bodies and axons/membranes.^[^ ^19^, ^20^ ^]^ Previous studies have shown that primary cilia are indeed absent in prostate cancer and that the development and progression of many tumors may be associated with the absence of primary cilia.^[^ ^11^ ^]^ We used Ac‐Tubulin and γ‐tubulin as markers to observe primary cilia formation. We detected DAPI, circ_0 005185, γ‐tubulin, and Acetylated‐tubulin (Ac‐Tubulin) in representative normal prostate, prostatic intraepithelial neoplasia (PIN), HSPC, and CRPC tumor samples by multispectral IF staining and continuous FISH experiments. The results showed that primary cilia were found with normal morphology in normal and PIN tissues, but cilia with significantly shortened length and abnormal morphology in HSPC and CRPC tissues. In addition, the fluorescence intensity of circ_0 005185 in CRPC tissue was significantly decreased (**Figure** **5**A). Immunofluorescence images from normal prostate epithelial cells (RWPE1) and CRPC cells (DU145) indicated that normal ciliary morphology can be observed in RWPE1 cells, while no such structures are present in DU145 cells (Figure S2A, Supporting Information). Immunofluorescence revealed the absence of primary cilia in the control DU145 and 22RV1 cell lines, whereas overexpression of circ_0 005185 promoted the formation of primary cilia in prostate cancer cell lines (Figure 5B). Western blotting results showed that the level of Ac‐Tubulin protein was significantly increased after the overexpression of circ_0 005185 (Figure S2B, Supporting Information). We grouped patients with prostate cancer in TCGA based on the expression of RAB8A. Subsequently, Gene Set Enrichment Analysis (GSEA) was performed on two groups of patients with high and low RAB8A expression. The results revealed significant differences between the two groups in terms of the Hh signaling pathway and androgen response (Figure 5C,D). Therefore, circ_0 005185 may be involved in the Hh signaling pathway and the androgen response through RAB8A. Subsequently, we used siRNAs to knock down RAB8A in the DU145 and 22RV1 cell lines (Figure S2C, Supporting Information). CCK8 and colony formation assays demonstrated that knocking down RAB8A and using SAG, an activator of the Hh signaling pathway, rescued the reduced viability of prostate cancer cells caused by the overexpression of circ_0 005185, indicating that circ_0 005185 may influence cellular functions through RAB8A and the Hh signaling pathway (Figure S2D,E, Supporting Information). Subsequently, we examined changes in the expression of key activators and repressors of the Hh signaling pathway. After overexpressing circ_0 005185, the level of P‐SMO and GLI1, a key activator in the Hh signaling pathway, remained unchanged, whereas the level of the truncated repressor form of GLI3 (GLI3R) increased, with no significant change observed in the full‐length form of GLI3F. In contrast, knocking down RAB8A decreased the formation of GLI3R (Figures 5E,F and S2F,G, Supporting Information). Additionally, after overexpressing circ_0 005185, we examined the expression of the Hh signaling pathway target genes CCND1 and c‐MYC, as well as the downstream target genes KLK3 and TMPRSS2 of the AR signaling pathway. The results illustrated that overexpression of circ_0 005185 inhibited the activation of downstream target genes in the Hh and AR signaling pathways, while knockdown of RAB8A rescued this effect (Figures 5G–I and S2H, Supporting Information). Research has suggested that the GLI family may regulate AR activity by interacting with AR.^[^ ^13^ ^]^ Therefore, we examined the protein levels of AR in control and circ0005185‐ OE cells in C4‐2 and 22RV1 cell lines. The results showed that overexpression of circ_0 005185 did not affect AR protein levels (Figure S2I, Supporting Information). Subsequently, we transfected cells with plasmids containing ARE and assessed AR activity through a luciferase assay. The results demonstrated an increase in AR activity after treatment with SAG, while overexpression of circ_0 005185 inhibited AR activity (Figure 5J). Furthermore, we treated both control and circ_0 005185 OE cells with increasing concentrations of DHT to measure AR activity. The results indicated that AR activity in circ_0 005185 OE cells was lower than in control cells, and following DHT treatment, AR transcriptional activity increased (Figure 5K). These findings suggest that circ_0 005185 inhibits AR activity rather than suppressing the AR protein level. In addition, activation of the Hh signaling pathway by SAG led to an increase in AR transcriptional activity, which was attenuated by overexpression of circ_0 005185. This indicates that circ_0 005185 suppresses AR activity by inhibiting the Hh signaling pathway.

### 2.6 Circ_0005185 Inhibits the Progression of Prostate Cancer In Vivo

**Figure 6:**
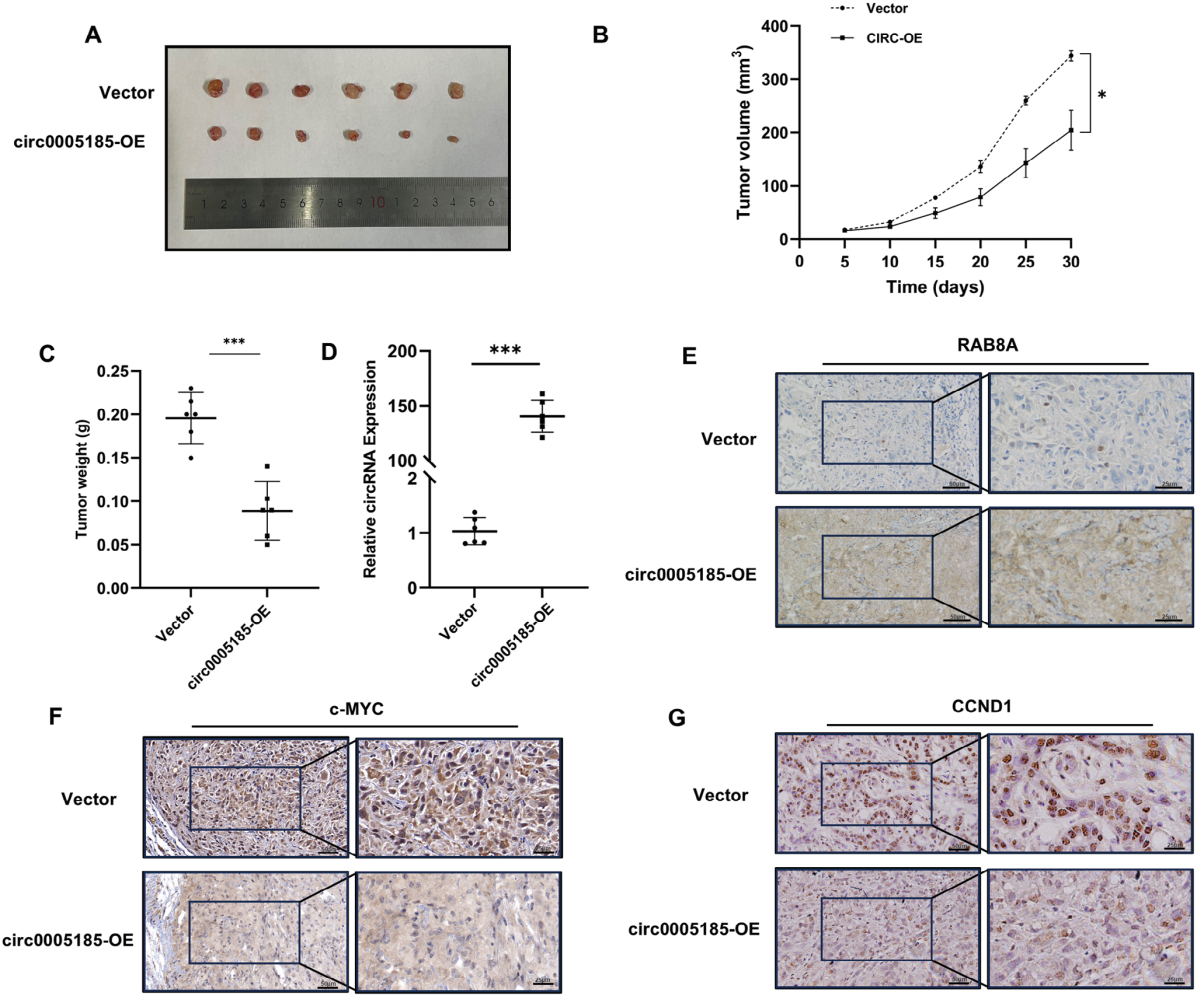
circ_0005185 inhibits the progression of prostate cancer in vivo. A) Images of subcutaneous xenografted tumor in the nude mice vector group and circ_0005185‐OE group. B) Tumor volume measured every 5 days. C) Weight of xenograft. D) qRT‐PCR showed that the expression of circ_0005185 in the tumors from the mice of the overexpression group was significantly higher than that in the control group. E–G) IHC results of RAB8A, CCND1, and c‐MYC in xenografts. Data are presented as the mean ± SD (^*^ *p* < 0.05; ^***^ *p* < 0.001; scale bar = 50 and 25 µm).

To investigate the anti‐tumor effect of circ_0 005185, a nude mouse xenograft model was established by injecting 4 × 10^6^ NC‐DU145 cells or circ0005185‐OE‐DU145 cells subcutaneously into the right axilla, and the tumor volume was measured every 5 days (**Figure** **6**A). The results illustrated that overexpression of circ_0 005185 significantly inhibited the growth of subcutaneous xenograft tumors in mice, with the weight and volume of tumors in the circ_0005185–overexpression group being significantly smaller than those in the control group (Figure 6B,C). The results of qRT‐PCR showed that the expression of circ_0 005185 in tumors from mice in the overexpression group was significantly higher than that in the control group (Figure 6D). In addition, immunohistochemical analysis revealed that in the tumors of mice overexpressing circ_0 005185, the abundance of RAB8A was increased, whereas that of c‐MYC and CCND1 was decreased (Figure 6E–G). These findings further validated the regulatory role of circ_0 005185 in RAB8A and the Hh signaling pathway in vivo.

## 3 Discussion

ADT is the cornerstone of endocrine treatment for prostate cancer and has been shown to reduce testosterone levels in the blood, decrease tumor burden, improve survival rates, and alleviate symptoms.^[^ ^24^ ^]^ However, most patients eventually develop resistance to ADT and progress to CRPC. The mechanisms underlying CRPC formation remain unclear, but factors such as AR mutations, AR amplification, abnormal androgen synthesis, and dysregulation of AR‐related gene expression all play crucial roles in its development and progression.^[^ ^25^ ^]^ CircRNAs regulate gene expression at multiple levels within organisms, with their primary mechanisms including, but not limited to, acting as miRNA sponges, modulating protein binding, regulating gene transcription, and encoding peptides.^[^ ^6^, ^26^ ^]^ To explore the potential mechanisms underlying the development and progression of CRPC, our preliminary study analyzed RNA sequencing data from four HSPC and three CRPC samples. This analysis, combined with data from public databases on circRNAs differentially expressed in high and low Gleason scores, as well as in enzalutamide‐sensitive and enzalutamide‐resistant prostate cancer cell lines, revealed that circ_0 005185 was consistently downregulated in CRPC samples, high Gleason score tumor tissues, and enzalutamide‐resistant cell line cohorts.

Further investigations, including RNA pull‐down and mass spectrometry experiments, confirmed the interaction between circ_0 005185 and OTUB1, as well as RAB8A. Overexpression of circ_0 005185 significantly increased RAB8A protein levels. OTUB1, a highly specific ubiquitin isopeptidase, can cleave ubiquitin from branched ubiquitin chains, resulting in deubiquitination.^[^ ^27^ ^]^ The activity and expression of OTUB1 are regulated by various factors. MicroRNAs significantly affect the progression and invasion of various cancers by targeting OTUB1 mRNA. For instance, miR‐542‐3p inhibits the migration, invasion, and proliferation of esophageal and colorectal cancer cells by targeting OTUB1.^[^ ^28^, ^29^ ^]^ The role of OTUB1 in cancer initiation and progression is not static. Studies have indicated that OTUB1 promotes bladder cancer development by stabilizing and deubiquitinating ATF6.^[^ ^30^ ^]^ Additionally, OTUB1 plays a crucial role in immune responses where it can inhibit AKT by removing ubiquitin, consequently disrupting its association with the cell membrane, which may impair the connection between AKT and PIP3.^[^ ^31^ ^]^ The inhibition of OTUB1 could stimulate robust immune‐related responses and serve as a clinical target for T‐cell therapy, suggesting that increasing OTUB1 expression may help suppress abnormal immune reactions. Recent research has also found that OTUB1 maintains low levels of PD‐L1 ubiquitination, indicating its potential as a target in immune evasion related to PD‐L1/PD‐1 therapy and in the immunosuppression of cancer cells.^[^ ^32^, ^33^ ^]^ However, the interaction between OTUB1 and RAB8A in prostate cancer cells, and the role of circRNAs in this context, has not been reported. The Co‐IP results demonstrated that OTUB1 can bind to RAB8A and that the overexpression of circ_0 005185 enhances this interaction. In addition, OTUB1 silencing led to reduced RAB8A protein levels.

RAB8A has long been recognized as a key factor in the polarization of epithelial cells and the targeting of extracellular components in neurons.^[^ ^34^ ^]^ Nachury et al. discovered that ciliary function is partially mediated by RAB8A, revealing the mechanisms between RAB8A and ciliary transport components. RAB8A enters primary cilia and promotes the extension of the ciliary membrane.^[^ ^35^ ^]^ Disruption of RAB8A function in zebrafish has been shown to inhibit ciliogenesis.^[^ ^36^ ^]^ As small GTPases, the activity of RAB8A can be regulated by GTPase‐activating proteins (GAPs). C9orf72 and its binding partner SMCR8 form a GTPase‐activating protein complex, which has been shown in vitro to regulate membrane transport and autophagy by acting as a GAP for RAB8A and RAB11A. Additionally, it has been discovered that the C9orf72‐SMCR8 complex negatively regulates primary cilium formation and Hh signaling by interacting with RAB8A.^[^ ^37^, ^38^, ^39^ ^]^ In this study, we observed post‐translational modifications of the key protein RAB8A involved in primary ciliogenesis. We found that circ_0 005185 acts as a scaffold, facilitating the binding between the deubiquitinating enzyme OTUB1 and RAB8A and mediating their deubiquitination process, thereby stabilizing the protein levels of RAB8A and promoting primary cilium formation.

Primary cilia are microtubule‐based organelles extending from the cell membrane that function as antennae to sense extracellular signals.^[^ ^14^ ^]^ Recent research has elevated this previously underappreciated organelle to the forefront of developmental biology studies and has highlighted its importance in developmental biology and human diseases such as cancer. Epithelial cells typically possess primary cilia. However, a reduction in primary cilia relative to adjacent normal tissue has been observed in tumor cells from renal cell carcinoma, breast cancer, melanoma, pancreatic cancer, and prostate cancer.^[^ ^40^, ^41^ ^]^ Although primary cilia formation is associated with the cell cycle, studies have shown that the loss of primary cilia is not a direct result of altered cell proliferation rates.^[^ ^15^ ^]^ Instead, ciliogenesis dysfunction in cancer might be due to other mechanisms, such as the loss of genes required for ciliogenesis, potentially caused by mutations or genomic instability. Primary cilia are involved in several signaling pathways crucial for disease development, including the Hh, Wnt, and platelet‐derived growth factor pathways.^[^ ^15^, ^42^, ^43^ ^]^ Notably, primary cilia are most closely related to the Hh signaling pathway, with ligand‐receptor interactions occurring within the ciliary structure.

Elevated Hh pathway activity is associated with various cancers, including prostate cancer.^[^ ^44^ ^]^ The Hh signal is typically inhibited by the tumor suppressor Ptch1, which suppresses the function of the proto‐oncogene Smo, a central activator of this pathway. The binding of the Hh ligand to PTCH1 relieves its inhibition of Smo, allowing Smo to induce downstream GLI activators and suppress the formation of GLI repressors.^[^ ^45^ ^]^ Upon phosphorylation by glycogen synthase kinase‐3β (GSK‐3β), protein kinase A (PKA), and CK1, GLI3 is cleaved into its repressor form, GLI3R, triggered by cyclic AMP.^[^ ^46^ ^]^ This action leads to the removal of the transcriptional activation domain of GLIs, allowing GLI3R to translocate to the nucleus and act as a repressor. Mutations in PTCH1 leading to loss of function have been linked to the upregulation of GLI1 and GLI2 in several cancers.^[^ ^47^, ^48^ ^]^ Recent studies have shown that cilia not only play a crucial role in Hh signal transduction but also negatively regulate this pathway. Genetic analyses have indicated that cilia are necessary for the proteolysis of Gli3 into the repressor form Gli3R.^[^ ^49^ ^]^ Therefore, cilia play both positive and negative regulatory roles in the Hh pathway. The role of primary cilia loss and Hh pathway activation in CRPC remains unclear. Research has shown that CRPC is associated with the reactivation of AR activity in tumor cells and the restoration of tumor androgen levels through AIS. PCa cells have been demonstrated to possess steroidogenic capabilities in vitro. In primary or metastatic prostate tumors undergoing ADT, stromal cells can stimulate AIS activation of AR through paracrine Hh signaling within the tumor microenvironment. Therefore, inhibiting the Hh signaling pathway may help target AIS originating from stromal cells of prostate tumors, thereby delaying the development of CRPC after ADT.^[^ ^18^ ^]^ Another study showed that the use of the Hh signaling pathway inhibitor cyclopamine suppressed the expression of endogenous androgen‐regulated genes in prostate cancer cells. Similarly, reducing the expression of Smo using siRNA also co‐inhibited the expression of androgen‐induced KLK2 and KLK3 without affecting AR mRNA or protein expression. Cyclopamine also blocked the growth of androgen‐dependent parental LNCaP cells and cell growth was restored when supplemented with androgens in the presence of cyclopamine.^[^ ^13^ ^]^ Our research showed overexpression of circ_0 005185 increased RAB8A levels in prostate cancer cells, thereby promoting ciliogenesis and inhibiting Hh signaling pathway activation. Therefore, the loss of circ_0 005185 may be a factor contributing to the absence of primary cilia in prostate cancer. Moreover, the loss of primary cilia could hinder the synthesis of the Hh pathway repressor GLI3R, ultimately leading to aberrant activation of the androgen receptor in prostate cancer cells. In this study, we also verified that activation of the Hh signaling pathway does not influence the protein levels of AR but rather promotes prostate cancer progression by enhancing AR activity. This effect may be related to the interaction between the GLI family and AR. We speculate that GLI3R inhibits AR activity by binding to AR, thereby suggesting that circ_0 005185 modulates AR activation by regulating the levels of GLI3R.

In recent years, although several circRNA drugs have started to enter clinical research stages, the clinical application of circRNA drugs is still in its infancy. Like many other cancer drugs, circular RNA drugs also face the challenge of developing efficient and safe methods for delivering circRNA or its modulators to tumor sites. Existing technologies have achieved the engineering and synthesis of target circRNA with endogenous expression in vitro, which can be delivered to animals using systems like Lipid NanoParticle (LNP), enabling precise, rapid, and controllable expression/overexpression of the desired circRNA.^[^ ^50^ ^]^ Therefore, we believe that various nano‐carrier drug delivery systems, including liposomes and other lipid‐based nanostructures, inorganic nanoparticles, and polymer nanoparticles, could potentially be utilized. Ligand modifications on these nanoparticles for selective targeting of PC cells can promote the internalization of cancer cells through receptor‐mediated endocytosis. For example, PSMA‐targeted delivery systems and LHRH‐targeted delivery systems.^[^ ^51^ ^]^

We acknowledge that this study has some limitations that warrant discussion. First, although the literature has confirmed the loss of cilia in pre‐invasive and invasive prostate cancer, further research is needed to explore their formation in various stages of prostate cancer, especially in CRPC. Additionally, this study is fundamental research exploring the function of circ_0 005185. Currently, there is limited direct clinical evidence supporting the effectiveness of targeting circ_0 005185 in humans. Therefore, we hope to further develop the clinical application research of circ_0 005185 in subsequent studies to potentially improve the prognosis of prostate cancer patients.

## 4 Conclusion

In this study, we discovered that circ_0 005185 was consistently lowly expressed in high Gleason score tumor tissues, CRPC samples, and enzalutamide‐resistant cell lines. Additionally, RNA pull‐down assays combined with mass spectrometry revealed that circ_0 005185 interacts with RAB8A and OTUB1. Notably, the overexpression of circ_0 005185 led to a significant increase in the protein levels of RAB8A. Furthermore, our findings indicated that circ_0 005185 overexpression promoted the formation of primary cilia and inhibited the activation of the Hh signaling pathway by regulating the production of GLI3R. Importantly, the knockdown of RAB8A diminished the increase in GLI3R associated with circ_0 005185 overexpression. Based on these observations, we hypothesized that circ_0 005185 mediates the deubiquitination of RAB8A by facilitating the binding of OTUB1 to RAB8A. This mechanism subsequently regulates the formation of primary cilia and the activation of the Hh signaling pathway, ultimately influencing the progression and drug resistance of prostate cancer.

## 5 Experimental Section

### Data Collection

The circRNA sequencing data of 10 patients with prostate cancer were obtained from GSE113153, while the circRNA sequencing data of enzalutamide‐resistant (high and low) cell lines and control cells were sourced from GSE118959. Three CRPC and four HSPC tumor tissue samples were prepared for RNA sequencing to identify differentially expressed circRNAs. CRPC samples were obtained from patients who underwent transurethral resection of the prostate (TURP). HSPC samples were collected from patients with pT3N0M0 stage disease who had radical prostatectomy (RP). Samples were sequenced by the Shanghai Biotechnology Corporation (Shanghai, China). To perform Gene Set Enrichment Analysis (GSEA) in a large prostate cancer cohort, RNA‐seq data of 493 prostate cancer samples were extracted from TCGA.

### Clinical Sample Collection

38 pairs of prostate cancer and para‐cancer samples were obtained from patients who underwent RP at Zhongshan Hospital, Fudan University between 2021 and 2023. CRPC tumor tissue samples were procured from four patients with an advanced pathological stage of pT4N 1M1 who exhibited non‐responsiveness to ADT. For the three patients with CRPC, we administered palliative TURP, with the primary objective of alleviating bleeding and obstructive symptoms and thereby enhancing their overall quality of life. The pathological diagnosis of each tumor tissue sample was independently verified and confirmed by two experienced pathologists, ensuring the utmost accuracy and reliability. The selection of tumor blocks adhered to the rigorous methodology outlined in one of our previously published studies, further reinforcing the scientific rigor of our investigation.^[^ ^52^ ^]^ This study was approved by the Ethics Committee of Zhongshan Hospital, Fudan University (No: B2020‐351R), and all patients provided written informed consent.

### Cell Lines and Cell Culture

The LNCaP, DU145, C4‐2, PC3, 22RV1, and RWPE1 cell lines were purchased from the Chinese Academy of Sciences Cell Bank. LNCaP, C4‐2, 22RV1, PC‐3, and RWPE1 cells were cultured in RPMI‐1640 medium (Corning Cellgro, Manassas, VA, USA), DU145 cells were cultured in Dulbecco's modified Eagle's medium (Corning Cellgro, Manassas, VA, USA). All media were supplemented with 10% fetal bovine serum (FBS; Gibco, Gaithersburg, MD, USA) and an antibiotic cocktail of 100 U mL^−1^ penicillin and 100 µg mL^−1^ streptomycin (HyClone, Logan, UT, USA). Cells were cultured at 37 °C in an atmosphere containing 5% CO_2_.

### Quantitative Real‐Time PCR (qRT‐PCR)

Total RNA from tissues and cells was extracted using TRIzol UP (TransGen Biotech, Beijing, China). CircRNA cDNA libraries were constructed using the PrimeScript RT kit and gDNA Eraser Kit (TaKaRa Biotech, Dalian, China). Primers covering the loop‐forming site of hsa_circ_0005185 were designed, and qRT‐PCR was performed with 18S RNA as a control using HieffqPCR SYBRGreen Master Mix (Yessen Biotech, Shanghai, China). The data were analyzed by the relative quantification (2‐ΔΔCT) method. The primers are described in Supplemental Table S2 (Supporting Information).

### Western Blot

Cells were lysed with RIPA lysis buffer (Beyotime, Shanghai, China), and total proteins were extracted, before quantifying the protein concentrations using a BCA kit (Beyotime, Shanghai, China). Protein samples were separated by SDS‐PAGE and transferred to polyvinylidene difluoride membranes (Merck‐Millipore, Burlington, MA. USA). The membranes were blocked with 5% skim milk powder for 2 h and then incubated with primary antibodies at 4 °C overnight. The membranes were then washed with tris‐buffered saline Tween‐20 (TBST) and then incubated with the secondary antibody at room temperature for 1.5 h. The antibodies used in this study are listed in Supplementary Table S2 (Supporting Information).

### RNase R Treatment

The RNA samples were incubated with 6 U µg^−1^ RNase r at 37 °C for 90 min, and after reverse transcription, the target fragments were amplified by PCR using divergent and convergent primers, and the expression of each target fragment was detected by 1% agarose gel electrophoresis or qRT‐PCR. The primers used in this study are listed in Supplementary Table S2 (Supporting Information).

### Cell Transfection

For gene overexpression and knockdown experiments, cells were seeded on 6‐cm plates and transfected with oligonucleotides using Lipofectamine 2000 (Thermo Fisher Scientific, Boston, MA, USA) according to the manufacturer's instructions. For stable expression, the lentiviral vector containing the desired gene, together with the packing plasmid pSPAX2 and envelope plasmid pMD2.G, were transfected into HEK293T cells at a ratio of 4:3:1. The supernatants containing lentiviral particles were collected and used to infect EOC cell lines with polybrene (8 µg mL^−1^). Puromycin (2 µg mL^−1^) was added to select positive cells. A lentivirus vector expressing hsa_circ_0005185 and its matched negative control were constructed by Lingke Biotechnology Co., Ltd. (Shanghai, China) for the overexpression of circ_0 005185. OBiO Technology (Shanghai, China) provided three siRNAs for the knockdown of RAB8A and OTUB1 and their homological negative controls. Information about the siRNA sequences is presented in Supplementary Table S3 (Supporting Information).

### Compounds

Enzalutamide, MG132, and SAG were acquired from MedChemexpress (USA) and were proportionally dissolved in DMSO. Dihydrotestosterone (DHT) was obtained from Aladdin (China) and dissolved in EtOH in proportion.

### Actinomycin D Assay

Cells were inoculated at a density of 70–80% in 6‐well plates (Corning, Incorporated, New York, USA), and 24 h later, the cells were exposed to 2 µg mL^−1^ actinomycin D (MedChemExpress, Monmouth Junction, NJ, USA) and collected at the indicated time points (0, 6, 12, and 18 h). RNA stability was analyzed using qRT–PCR.

### Cell Viability

For the cell proliferation assay, cells (2000 cells/well) were inoculated in 96‐well cell culture plates and cultured using the serum‐containing medium. The cells were incubated with CCK‐8 reagent (Yeason, Shanghai, China) for 2 h after 0, 24, 48, 72, and 96 h of cell apposition. The absorbance was measured at 450 nm using an enzyme marker. For the cytotoxicity assay, 5000 cells per well were inoculated in 96‐well plates and treated with different concentrations of compounds after 24 h. Cell viability was detected using the CCK‐8 assay. For the colony formation assay, 1000 cells/well were inoculated in 6‐well plates and cultured in 10% FBS for 12 days. Then, the cells were fixed with 4% paraformaldehyde, and the number of clones was recorded after staining with crystal violet.

### Wound Healing and Transwell Migration Assays

The cells were evenly seeded onto a six‐well cell culture plate. Once the cell density reached 90%, a line was drawn on the plate's bottom with a 10‐microliter pipette tip. The plate was then incubated in a serum‐free medium for 36 h. Microscope images of the scratches were taken to observe the extent of healing. For the Transwell assay, 24‐well Transwell chambers were used, each containing an 8‐µm polycarbonate membrane (Corning, NY). The upper chamber was filled with serum‐free medium, whereas the lower chamber contained 10% FBS medium. The upper chamber was filled with 50000 cells and incubated at 37 °C with 5% carbon dioxide for 48 h. Subsequently, the cells were fixed in methanol, stained with crystal violet, and photographed under a microscope.

### In Situ Hybridization (ISH)

TMA was dewaxed in xylene and rehydrated sequentially with 100%, 95%, 85%, and 75% alcohol. Then, the tissues were hybridized with a specific digoxin‐labeled circ_0 005185 probe (Servicebio, Wuhan, China).

Fluorescence in situ hybridization (FISH) assay

Specific FISH probes targeting hsa_circ_0005185 were designed by GenePharma (Shanghai, China) and detected using a fluorescence in situ hybridization kit (GenePharma, Shanghai, China) according to the manufacturer's instructions. All stained cells were examined and photographed using a Zeiss LSM880 confocal fluorescence microscope (Carl Zeiss, Jena, Germany).

### Immunofluorescence Assay

Cells were cultured in 24‐well plates. The cells were then fixed in methanol for 15 min and treated with 0.5% Triton X‐100 for 10 min at 4 °C. The cells were then incubated in 5% bovine serum albumin (BSA) at 4 °C overnight. Subsequently, the BSA solution was aspirated, and the samples were treated with primary antibody (1:200) overnight. After incubation, the samples were washed three times with phosphate buffer Tween 20 (PBST), before mixing with a secondary antibody (1:200) and incubating for 1.5 h at room temperature. The washing process was repeated three more times with PBST. The samples were blocked with DAPI Fluoromount‐G (SouthernBiotech, Birmingham, AL, USA). We performed multispectral immunofluorescence (IF) staining on tissue sections. In brief, the slides were heated, deparaffinized with xylene, and rehydrated through graded alcohols. After antigen retrieval and blocking, primary antibodies were applied and incubated overnight at 4 °C. The secondary antibody used was a polymeric horseradish peroxidase (HRP). The slides were washed, followed by signal amplification using tyramide signal amplification (TSA) (TSA Kit; Servicebio, Wuhan, China). Subsequently, the slides were microwaved to remove the primary and secondary antibodies, washed, and then blocked again with blocking solution. Finally, a secondary antibody and DAPI were applied. The antibodies used are listed in Supplementary Table S4 (Supporting Information).

### RNA Pull‐Down and Mass Spectrometry Analyses

Cells were lysed with RIPA lysis buffer (Leagene Biotechnology, Beijing, China) and incubated with the biotin‐labeled circ_0 005185 probe after binding to streptavidin magnetic beads. The biotin‐labeled probe targeting the circ_0 005185 junction site was synthesized by GenePharma (Shanghai, China). Proteins bound to circ_0 005185 were extracted using a PierceTM Magnetic RNA Protein Pull‐Down Kit (20164Y, Thermo Fisher Scientific, Boston, MA, USA). Finally, the interacting proteins were identified by silver staining using a rapid silver staining kit (P0017S, Beyotime, Shanghai, China) and analyzed by mass spectrometry (Applied Protein Technology, Shanghai, China).

### RNA‐Binding Protein Immunoprecipitation (RIP) Assay

Prostate cancer cells were treated with IP lysis buffer (Leagene Biotechnology, Beijing, China) and stored in lysis buffer at −80 °C overnight. Magna RIPTM RNA Binding Protein Immunoprecipitation Kit (17‐701, Merck KGaA, Darmstadt, Germany), OTUB1 primary antibody (Proteintech, Chicago, USA), and RAB8A primary antibody (Proteintech, Chicago, USA) were used to extract protein‐bound RNA, and IgG primary antibody was used as a control. A circRNA cDNA library was constructed using a PrimeScript RT kit and gDNA Eraser (TaKaRa Biotech, Dalian, China). qRT–PCR was performed to detect the RNA level of circ_0 005185, and western blotting was performed to detect the OTUB1 and RAB8A protein levels.

### Co‐Immunoprecipitation (Co‐IP) Assay

Prostate cancer cells were treated with IP lysis buffer (Leagene Biotechnology, Beijing, China), and Co‐IP experiments were performed using anti‐OTUB1 primary antibody, anti‐RAB8A primary antibody, and protein A/G immunoprecipitation magnetic beads (B23202, Bimake, Shanghai, China), with an anti‐IgG primary antibody used as the control. Sodium dodecyl sulfate‐polyacrylamide gel electrophoresis was used to detect protein expression.

### AR Activity Assay

AR activity was detected by luciferase assay. The 6 × AR response element (ARE) consensus sequence (GGGAACACAATGTTCCC) was cloned into the pCDH–NC–EF1‐PURO‐RLUC Lenti‐vector provided by Transheep in Shanghai, China. HEK293T cells were co‐transfected with ARE‐Reporter‐LUC and three lentiviral packaging vectors: pRSV‐Rev, pMDLg/pRRE, and pMD 2. G. The supernatants containing the lentivirus were collected and concentrated. Subsequently, the C4‐2 and 22RV1 cells were coinfected with the ARE‐Reporter lentivirus, circ0005185‐OE lentivirus, and the corresponding control virus. Initially, the infected cells were plated in 24‐well plates and cultured in 10% charcoal‐stripped fetal bovine serum (CSS) for 24 h. Following this, the cells were treated with 0.1, 0.2, and 0.4 nM DHT or EtOH for an additional 24 h. The luciferase activity was quantified using a Dual‐Luciferase assay kit from Yeason in Shanghai, China. Furthermore, the luciferase activity of C4‐2 and 22RV1 cells in both the control group and the circ0005185‐OE group was assessed. Subsequently, the luciferase activity of cells within these groups was reevaluated after SAG treatment.

### Immunohistochemistry

Immunohistochemistry was performed with the GTVision + Detection System/Mo&Rb Immunohistochemistry Kit (GK500710, GeneTech, Shanghai, China) using primary antibodies to quantify protein levels in tissues according to the manufacturer's instructions. The antibodies are listed in Supplementary Table S4 (Supporting Information).

### Subcutaneous Tumor Xenograft Assay

Twelve male BALB/c nude mice (5 weeks old) were obtained from Gempharmatech Ltd. (Nanjing, China). Mice were housed in a pathogen‐free laminar flow cabinet and given 1 week to acclimate. The mice were then divided into two groups (6 mice per group) and injected with 4 × 10^6^ NC‐DU145 cells or circ_0005185‐OE‐DU145 cells subcutaneously into the right axilla. The tumor size was measured every 5 days using Vernier calipers. The tumor volume was calculated as V = (length × width^2^)/2. After 30 days, following animal ethics, the mice were euthanized, and the tumors were extracted to measure their size and weight.

### Statistical Analysis

All experiments were repeated independently at least three times to determine the accuracy of the experimental outcomes. GraphPad Prism 9 software was used for graph construction, and ImageJ was used to analyze the experimental data. The data measurements are expressed as the mean ± standard deviation (SD), where comparisons between two groups were conducted using Student's t‐test and comparisons between three or more groups were conducted using two‐way ANOVA.

### Ethics Approval and Consent to Participate

This study was approved by the Ethics Committee of Zhongshan Hospital, Fudan University (Approval No: B2020‐351R), and written informed consent was obtained from all patients. All animal experiments and protocols were reviewed and approved by the Animal Care and Use Committee of Shanghai University.

## Conflict of Interest

The authors declare no conflict of interest.

## Author Contributions

A.F., Y.Z., and Y.Li contributed equally to this work. Wei Chen and Zhongliang Ma performed the study concept and design; A. F. and Y. Z. developed the methodology and wrote, reviewed, and revised the manuscript; A. F., Y. Z., and Y. L. acquired, analyzed, and interpreted the data, and performed statistical analysis; W. M. and F. W. provided technical and material support. All authors have read and approved the final paper.

## Supporting information



Supporting Information

## Data Availability

Research data are not shared.
