# Supplementary material for: Primary Cilia Formation Mediated by Hsa_Circ_0005185/OTUB1/RAB8A Complex Inhibits Prostate Cancer Progression by Suppressing Hedgehog Signaling Pathway
Source: Adv Sci (Weinh). 2025 Jan 9;12(8):2411675. doi: 10.1002/advs.202411675 (PMC11848605; doi:10.1002/advs.202411675)
Supplement: Supplementary file 1 — Supporting Information [file ADVS-12-2411675-s001.docx]

**Supporting Information**

Title: Primary Cilia Formation Mediated by Hsa_circ_0005185/OTUB1/RAB8A Complex Inhibits Prostate Cancer Progression by Suppressing Hedgehog Signaling Pathway

*Aoyu Fan, Yunyan Zhang, Yunpeng Li, Wei Meng, Fan Wu, Wei Pan, Zhongliang Ma^*^, Wei Chen^*^*

Aoyu Fan, Yunyan Zhang, and Yunpeng Li have contributed equally to this work.

Aoyu Fan, Yunyan Zhang, Yunpeng Li, Wei Chen

Department of Urology, Zhongshan Hospital, Fudan University, 200030 Shanghai, China

chen.wei3@zs-hospital.sh.cn

Wei Meng, Fan Wu, Wei Pan, Zhongliang Ma

Lab for Noncoding RNA and Cancer, School of Life Sciences, Shanghai University, 200444 Shanghai, China

zlma@shu.edu.cn

**
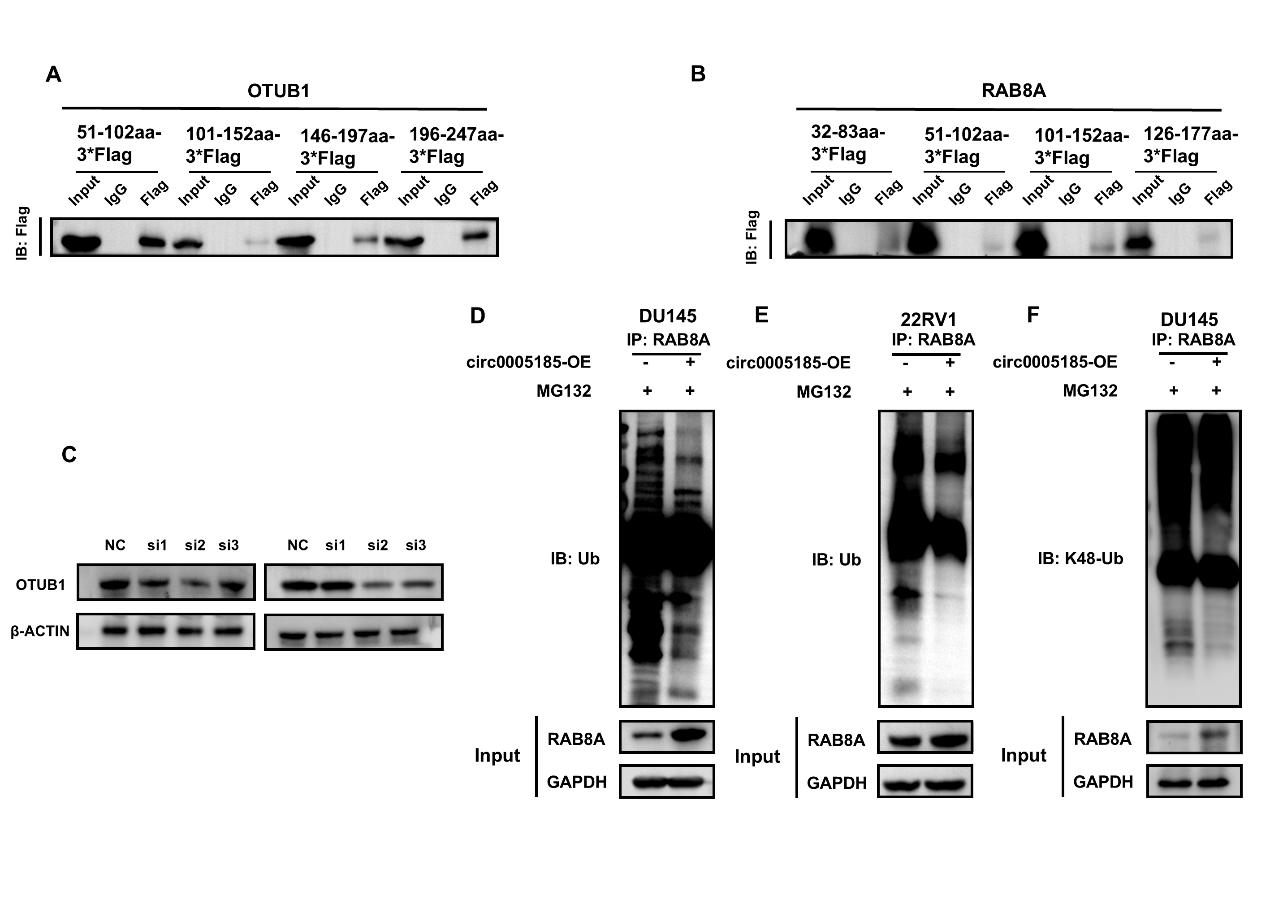
**

Figure S1

A–B) Western blot was used to detect the binding of Flag protein to magnetic beads. C) Western blot was performed to validate the knockdown efficiency of siRNAs targeting OTUB1. D-E) DU145 and 22RV1 cells in the control group and circ_0005185 overexpression group were treated with MG132. After purifying RAB8A protein by Co-IP, the ubiquitination level of the RAB8A protein was detected. F) DU145 and 22RV1 cells in the control group and circ_0005185 overexpression group were treated with MG132. After purifying the RAB8A protein by Co-IP, the ubiquitination level of RAB8A protein at the K48 site was detected using K48 site ubiquitination antibody.

**
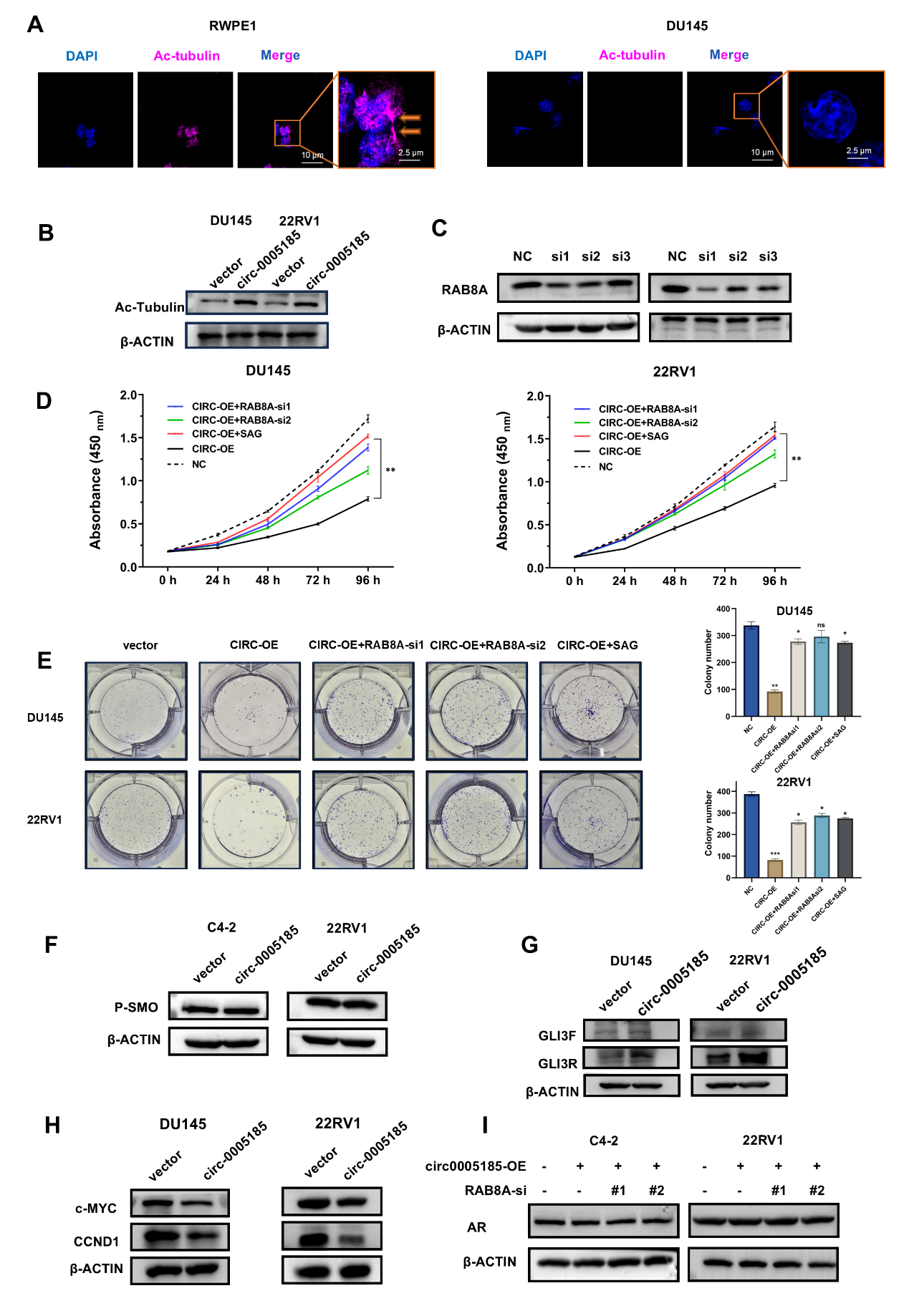
**

Figure S2

A) Immunofluorescence detection of DAPI (blue), Ac-tubulin (pink), and primary cilia (yellow arrows) from normal prostate epithelial cells (RWPE1) and CRPC cells (DU145). B) Western blotting results showed that the level of Ac-Tubulin protein was significantly increased after the overexpression of circ_0005185. C) Western blot was performed to validate the knockdown efficiency of siRNAs targeting RAB8A. D) CCK8 assays were conducted to assess the proliferation ability of DU145 and 22RV1 cells in the control and circ_0005185 overexpression groups. Knockdown of RAB8A and treatment with Smoothened Agonist (SAG) rescued the reduced proliferation capacity observed in the circ_0005185 overexpression group. E) Colony formation assays were used to evaluate the viability of DU145 and 22RV1 cells in the control group and circ_0005185 overexpression group. Similarly, knockdown of RAB8A and SAG treatment reversed the decreased cell viability in the circ_0005185 overexpression group. F) Western blot showed that overexpressing circ_0005185 didn’t affect the protein levels of P-SMO in the Hh signaling pathway. G) Western blot revealed an elevation in GLI3R following circ_0005185 overexpression. H) Overexpressing circ_0005185, the protein levels of downstream target genes CCND1 and c-MYC in the Hh signaling pathway were decreased. I) WB results showed that overexpression of circ_0005185 and knockdown of RAB8A in C4-2 and 22RV1 cells did not affect the protein level of AR. Data are presented as the mean ± SD (*P < 0.05; ***P* < 0.01; *** P < 0.001; ns, not significant).

**Supplementary Table** **S1: Correlation between circ_0005185 expression and clinicopathological characteristics in PCa**

|  | **High** | **Low** | **p.overall** |
| --- | --- | --- | --- |
|  | ***N=19*** | ***N=19*** |  |
| Age | 61.9 (5.13) | 65.1 (4.52) | 0.055 |
| T: |  |  | <0.001 |
| T2 | 17 (89.5%) | 5 (26.3%) |  |
| T3 | 2 (10.5%) | 14 (73.7%) |  |
| N: |  |  | 0.042 |
| N0 | 18 (94.7%) | 12 (63.2%) |  |
| N1 | 1 (5.26%) | 7 (36.8%) |  |
| Gleason_score: |  |  | <0.001 |
| 7 | 10 (52.6%) | 1 (5.26%) |  |
| 8 | 5 (26.3%) | 3 (15.8%) |  |
| 9 | 4 (21.1%) | 15 (78.9%) |  |

**Supplementary Table S2. Sequences of PCR primers and RNA pulldown probe.**

| **Category** | **Sequence (5'-3')** |
| --- | --- |
| hsa_circ_0005185 | Forward: CAATTCTACAGATTTGCTTA |
| hsa_circ_0005185 | Reverse: TTTGACAGACTGCACTACC |
| Divergent primer | Forward: TGGGTTCTGGACAGTTTG |
| Divergent primer | Reverse: TTGGACCATTGCTTCTTC |
| Convergent primer | Forward: GAAACAGAAATGAAAGCCCTCG |
| Convergent primer | Reverse: GGCAACTCAGATACATCAACAG |
| PRKD1 | Forward: AACAGAAATGAAAGCCCTCG |
| PRKD1 | Reverse: GCTTCTGTTGATTTGTCTTGGC |
| RAB8A | Forward: CAACGGCCTACTACAGGGG |
| RAB8A | Reverse: GGATGTTGTCGAAGGACTTCTC |
| CCND1 | Forward: GCTGCGAAGTGGAAACCATC |
| CCND1 | Reverse: CCTCCTTCTGCACACATTTGAA |
| c-MYC | Forward: GGCTCCTGGCAAAAGGTCA |
| c-MYC | Reverse: CTGCGTAGTTGTGCTGATGT |
| KLK3 | Forward: CACAGGCCAGGTATTTCAGGT |
| KLK3 | Reverse: GAGGCTCATATCGTAGAGCGG |
| TMPRSS2 | Forward: GTCCCCACTGTCTACGAGGT |
| TMPRSS2 | Reverse: CAGACGACGGGGTTGGAAG |
| GAPDH | Forward: AGAAGGCTGGGGCTCATTTG |
| GAPDH | Reverse: AGGGGCCATCCACAGTCTTC |
| hsa_circ_0005185 pulldown | TGCCCCAGGGCTAAGCAAATCTGTAGAAT（5'-3'） |

**Supplementary Table S3.** **Sequences for siRNAs transfection.**

| **Category** | **Sequence** |
| --- | --- |
| si-OTUB1-1 | 5'-GGAAGUCAACUCCAAUCA-3' |
| si-OTUB1-2 | 5'-AGGAGTATGCTGAAGATGACA-3' |
| si-OTUB1-3 | 5'-AGCGACTCCGAAGGTGTTAAC-3' |
| si-RAB8A-1 | 5'-CGGAACUGGAUUCGCAACAUUTT -3' |
| si-RAB8A-2 | 5′-CUCGAUGGCAAGAGAAUUAAATT -3′ |
| si-RAB8A-3 | 5′-UCGCCAGAGAUAUCAAAGCAATT -3′ |

**Supplementary Table S4.** **Primary antibodies.**

| **Antibody** | **Manufacturer** | **Catalog number** |
| --- | --- | --- |
| Anti-OTUB1 | Abcam | ab270959 |
| Anti-RAB8A | Abcam | ab241061 |
| Anti-GLI1 | Abcam | ab134906 |
| Anti-GLI3 | Abcam | ab307714 |
| Anti-AR | Abcam | Ab108341 |
| Anti-CCND1 | Abclonal | A1301 |
| Anti-P-SMO | Abclonal | A3274 |
| Anti-c-MYC | Proteintech | 3D9C12 |
| Anti-γ-tubulin | Abclonal | A9657 |
| Anti-Ac-tubulin | Proteintech | 7E5H8 |
| Anti-β-actin | Proteintech | 20536- |
| Anti-GAPDH | Abcam | ab9485 |
| Ubiquitin | CST | 3936T |
| K48-linkage specific polyubiquitin | CST | 8081S |
| K63-linkage specific polyubiquitin | CST | 5621S |
